# Choroidal thickness in relation to urinary albumin excretion rate in type 2 diabetes mellitus without retinopathy

**DOI:** 10.1186/s40942-021-00332-6

**Published:** 2021-10-16

**Authors:** Doaa Maamoun Ashour, Amany Abd El-Fattah El-Shazly, Randa Hesham Ali Abdelgawad, Mohamed Ibrahim Saleh

**Affiliations:** Ophthalmology Department, Faculty of Medicine, Ain Shams University, Cairo, Egypt

**Keywords:** Choroidal thickness, Diabetic retinopathy, Microalbuminuria, Urinary albumin excretion rate

## Abstract

**Background:**

To evaluate choroidal thickness (CT) in diabetic patients without diabetic retinopathy (DR) in relation to the urinary albumin excretion rate (UAER).

**Methods:**

This is a prospective case-control study that included a consecutive sample of 120 patients with type 2 diabetes without clinically evident DR and a group of 60 matched healthy controls. Diabetic patients were included in two groups according to their UAER (normoalbuminuria and microalbuminuria). Complete ophthalmological examination was performed followed by optical coherence tomography (SD-OCT) for retinal and choroidal assessment. Twenty-four-hour urine samples were collected for UAER and blood samples for HbA1c and serum creatinine were obtained.

**Results:**

The study included 180 eyes from 180 subjects in three groups. Patients with higher levels of albuminuria had a thinner choroid than normal controls, with decremental thinning as albuminuria progressed. Diabetics with normoalbuminuria showed no significant differences from controls. Choroidal thickness showed a significant moderate negative correlation with UAER (r  =  − 0.58, p  <  0.001). Multiple regression analyses for diabetic patients with microalbuminuria demonstrated that UAER is the most important determinant of subfoveal choroidal thickness (SFCT) (p  <  0.001).

**Conclusions:**

Decreased CT was significantly correlated with UAER in diabetic patients without retinopathy and otherwise normal kidney functions. This decrease in thickness might be a predictor of DR.

## Introduction

Diabetes is one of the greatest health problems worldwide, with an estimated global prevalence of 9.3% (463 million people) in 2019, with an expected increase to 10.9% (700 million) by 2045 [1]. More than 90% of diabetic patients suffer from type 2 diabetes mellitus (DM), which is a known cause of various microvascular and macrovascular complications that can significantly affect patient life [2]. Diabetic retinopathy (DR) is one of the complications that affects more than 35% of diabetic patients and is considered the leading cause of visual loss in working-age adults [3]. Thus, screening programs for early detection of DR have been widely adopted. However, with variable socioeconomic levels in different areas many diabetic patients are not undergoing regular screening until they present with late complications [4].

Various studies were conducted aiming to identify early predictors of DR progression [5]. One of the identified prognostic factors is microalbuminuria, which was identified as an early marker of generalized endothelial damage associated with chronic microvascular complications of diabetes [6]. Yearly, 5–10% of type 2 DM with microalbuminuria develop diabetic nephropathy, which is also associated with an increased risk of developing DR [7].

Previous clinical and histopathological studies have shown that vascular dysfunction in DM may affect both the choroidal and retinal vasculature. Several choroidal abnormalities have been reported in the eyes of diabetic patients including increased vascular tortuosity, obstruction of the choriocapillaris, and areas of non-perfusion or neovascularization [8–11].

With the continuous advancements in imaging techniques, detailed choroidal observation and measurements have become possible via new generations of optical coherence tomography (OCT) systems including enhanced depth imaging (EDI) mode in spectral-domain (SD-OCT), swept-source OCT, en face OCT, and optical coherence tomography angiography [12]. Using these machines, many studies have shown a significant difference in choroidal thickness (CT) of diabetic eyes compared to healthy eyes [13–16]. However, the findings of these studies were mostly controversial and inconsistent, especially in patients without DR [17–19].

Identification of an early predictor for DR via simple non-invasive imaging will be of utmost importance and will save many eyes. Knowing that microalbuminuria was previously identified as the earliest sign of diabetic nephropathy and an early predictor for DR, we conducted this research to study CT changes in the eyes of diabetic patients before evident diabetic changes and to compare these changes with the patients’ urinary albumin excretion rate (UAER). Aiming to identify the possibility of using choroidal thickness changes as an early predictor of DR.

## Methods

This is a prospective case-control study. The study protocol was approved by the Research Ethics committee of the Faculty of Medicine, Ain Shams University. The study strictly adhered to research ethics as stated in the Declaration of Helsinki.

Patients were recruited from the outpatient clinic at Ain Shams University Hospital, Cairo, Egypt. Diabetic patients who met the inclusion criteria were invited to participate (n  =  120). A group of healthy controls was included (n  =  60). Written informed consent was obtained from all participants.

Inclusion criteria included being diagnosed with type 2 DM for more than 5 years without any evidence of DR on dilated fundus examination. Exclusion criteria included a refractive error of more than  ±  3 diopters (D), pre-existing retinal diseases, uveitis, glaucoma, media opacities, previous ocular trauma, or any ocular surgery. Patients with axial length (AL) longer than 25 mm were excluded due to the previously identified negative correlation between AL and CT [20]. Patients were also excluded if they were smokers or having uncontrolled systemic hypertension (150/95 mm Hg) [19], systemic lupus erythematosus, anemia, leukemia, obstructive sleep apnea, or any neurodegenerative disease. Patients with albuminuria more than 300 mg/day and/or elevated serum creatinine, Blood Urea Nitrogen, or decreased estimated glomerular filtration (eGFR) rate in their records were excluded from this study.

Study participants were divided into three groups: the control group (group 1) included 60 healthy participants who were age and sex-matched to the patients. The recruited diabetic patients were subdivided into two groups according to their UAER as measured in sterile 24-h timed urine samples by immunoturbidimetry (Microlab; Ames, Tarrytown, NY). Normoalbuminuria was defined as UAER less than 30 mg/day, and microalbuminuria as UAER 30–300 mg/day [21]. Diabetic patients with normoalbuminuria were assigned to group 2. Group 3 included patients with microalbuminuria.

All participants underwent a careful history taking, review of their annual systemic work-up including their laboratory investigations, complete ophthalmic examination, including best-corrected visual acuity (BCVA), anterior segment examination, intraocular pressure (IOP), and dilated fundus examination. Retinal and choroidal imaging were performed using SD-OCT, Nidek RS-3000 Advance (Nidek, Gamagori, Japan). Finally, axial length (AL) was obtained (PacScan 300A; Sonomed Escalon Inc, New York, NY).

Blood samples were obtained for random blood sugar (RBS), serum creatinine, and HbA1c measurements. Estimated glomerular filtration rate (eGFR) was calculated using the Modification of Diet in Renal Disease (MDRD) formula [22].

Optical coherence tomography was performed after mydriasis using tropicamide 0.5% tropicamide eye drops (Mydriacyl 0.5%; Alcon Inc). Images were taken between 10 am and 12 pm to avoid diurnal variations [23]. Macular map was obtained, followed by choroidal imaging via the enhanced depth technique by approaching the OCT device closer to the eye until an inverted image with clear choroidal details and good signal strength was obtained. Macula 12 radial scans were obtained, and the sclerochoroidal interface was manually outlined in each scan. Image capture and manual editing were conducted by the same experienced OCT operator who was blinded from the diagnosis of the patients. All scans were revised for signal strength, centration, and accurate choroidal outlines between the outer boundaries of the retinal pigment epithelium and the sclero-choroidal interface prior to inclusion. Finally, a choroidal thickness map was generated by the incorporated software using the ETDRS chart including subfoveal choroidal thickness (SFCT) in the innermost 1 mm circle, inner 3 mm circle, and outer 6 mm circle. The 3 mm and 6 mm circles were divided into superior, inferior, nasal, and temporal quadrants.

Axial length measurements were obtained after previous noncontact imaging using A-scan after topical corneal anesthesia (0.4% benoxinate HCl eye drops). Five axial length (AL) measurements were obtained to calculate the average value.

## Statistical analysis

For data analysis, the Statistical Package for Social Sciences version 15.0 (SPSS© v. 15.0, SPSS Inc., Chicago, IL, USA) was used. Quantitative data were presented as mean  ±  standard deviation (SD). Gender differences were done using the Chi-square test. Multiple groups mean of parametric data sets were compared using a one-way analysis of variance (ANOVA) test. Further analysis with Tukey’s honestly significant difference (HSD) posthoc test if an overall significance was found. Pearson’s correlation and regression analyses were done to evaluate the different factors that influence choroidal thickness. p values  <  0.05 were considered statistically significant.

## Results

### Baseline characteristics

In this study, a total of 180 eyes (one eye from each patient randomly selected) of 180 subjects were included. Sixty in each group. The age ranged from 40 to 63 years. They were 85 males and 95 females without a statistically significant difference between the various groups. Male/female ratio was 26/34 in group 1, 28/32 in group 2, and 31/29 in group 3 (χ^2^  =  0.87 and p  =  0.833).

Slit-lamp and fundus examinations did not reveal abnormalities in all patients. There were no significant differences between the three groups in age, IOP, BCVA, and spherical equivalent (SE) as determined using one-way ANOVA (Table 1). All the included subjects had normal serum creatinine and eGFR with no statistically significant difference between the study groups (Table 1). One-way ANOVA showed that HbA1c and UAER were higher in diabetics than in controls. Post hoc test revealed that the HbA1c and UAER were statistically significantly lower in group 1 than in group 2, and both were lower in group 2 than in group 3. (Table 1).

**Table 1:** Clinical and demographic characteristics of the studied groups

	Group 1	Group 2	Group 3	F	p	Post hoc test		
	Mean ± SD	Mean ± SD	Mean ± SD			1 vs 2	1 vs 3	2 vs 3
Age (years)	47.31 ± 4.71	48.45 ± 4.75	48.98 ± 4.99	1.87	0.157	0.404	0.143	0.817
Duration of DM (years)	–	7.96 ± 2.16	8.42 ± 2.35	1.29	0.259	–	–	0.259
HBA1c (%)	5.75 ± 0.42	8.08 ± 0.88	11.53 ± 1.20	637.14	< 0.001	< 0.001	< 0.001	< 0.001
RBS (mg/dl)	182.93 ± 28.12	225.85 ± 37.00	309.70 ± 48.23	166.79	< 0.001	< 0.001	< 0.001	< 0.001
BCVA (LogMAR)	0.00 ± 0.00	0.00 ± 0.00	0.00 ± 0.00	–	–	1.000	1.000	1.000
IOP (mm Hg)	13.85 ± 1.62	13.43 ± 1.42	13.75 ± 1.63	1.03	0.358	0.326	0.702	0.803
SE (D)	− 0.26 ± 0.52	− 0.30 ± 0.39	− 0.35 ± 0.39	0.65	0.523	0.862	0.490	0.809
AL (mm)	23.82 ± 0.70	23.96 ± 0.66	23.98 ± 0.51	1.07	0.344	0.470	0.372	0.984
UAER (µg/min)	11.94 ± 2.71	13.89 ± 42.08	115.71 ± 42.08	352.35	< 0.001	0.915	< 0.001	< 0.001
Serum creatinine (mg/dL)	0.78 ± 0.12	0.79 ± 0.10	0.77 ± 0.11	0.49	0.612	0.873	0.873	0.582
eGFR (mL/min/1.73 m^2^)	100.78 ± 5.83	99.82 ± 5.16	100.47 ± 5.31	0.48	0.616	0.599	0.948	0.790

*HBA1c* glycosylated haemoglobin; *RBS* random blood sugar; *CCT* central corneal thickness; *BCVA* best corrected visual acuity; *IOP* intraocular pressure; *SE* spherical equivalent; *AL* axial length; *UAER* urinary albumin excretion rate; *eGFR* estimated glomerular filtration rate calculated using the MDRD (modification of diet in renal disease) formula

### Choroidal thickness

One-way ANOVA revealed that CT was significantly thinner in diabetic patients compared to control subjects in 9 locations (Table 2). Post hoc (Tukey’s HSD) showed that CT showed insignificant differences between the normoalbuminuric patients and the controls in the 9 sectors, while, in the nine sectors, the CT of group 3 was statistically lower than those of groups 1 and 2. (Table 2).

**Table 2:** Choroidal thickness (CT) of the studied groups

Variable (µm)	Group 1	Group 2	Group 3	F	P	Post hoc test		
	Mean ± SD	Mean ± SD	Mean ± SD			1 vs 2	1 vs 3	2 vs 3
SFCT	293.48 ± 28.51	285.87 ± 20.30	270.82 ± 14.90	16.55	< 0.001	0.141	< 0.001	< 0.001
SIM	285.48 ± 26.08	278.88 ± 21.51	266.62 ± 22.41	10.03	< 0.001	0.273	< 0.001	0.013
IIM	287.47 ± 36.19	279.02 ± 24.93	261.67 ± 36.36	9.86	< 0.001	0.218	< 0.001	0.019
TIM	277.03 ± 28.48	273.72 ± 35.27	259.10 ± 22.83	6.36	0.002	0.809	0.003	0.019
NIM	280.27 ± 21.96	275.88 ± 31.45	261.27 ± 25.38	8.42	< 0.001	0.638	< 0.001	0.008
SOM	268.85 ± 19.00	263.48 ± 18.55	250.20 ± 23.10	13.40	< 0.001	0.319	< 0.001	0.001
IOM	270.32 ± 16.88	266.32 ± 14.67	254.90 ± 22.54	11.43	< 0.001	0.458	< 0.001	0.002
TOM	272.65 ± 23.94	265.10 ± 21.35	251.12 ± 18.05	15.86	< 0.001	0.129	< 0.001	0.001
NOM	270.87 ± 21.29	267.33 ± 20.82	255.68 ± 22.07	8.27	< 0.001	0.638	< 0.001	0.009

*SFCT* subfoveal choroidal thickness; *SIM* superior of the inner macula (CT); *IIM* inferior of the inner macula (CT); *TIM* temporal of the inner macula (CT); *NIM* nasal of the inner macula (CT); *SOM* superior of the outer macula (CT); *IOM* inferior of outer macula (CT); *TOM* temporal of the outer macula (CT); *NOM* nasal of the outer macula (CT)

**Fig. 1:**
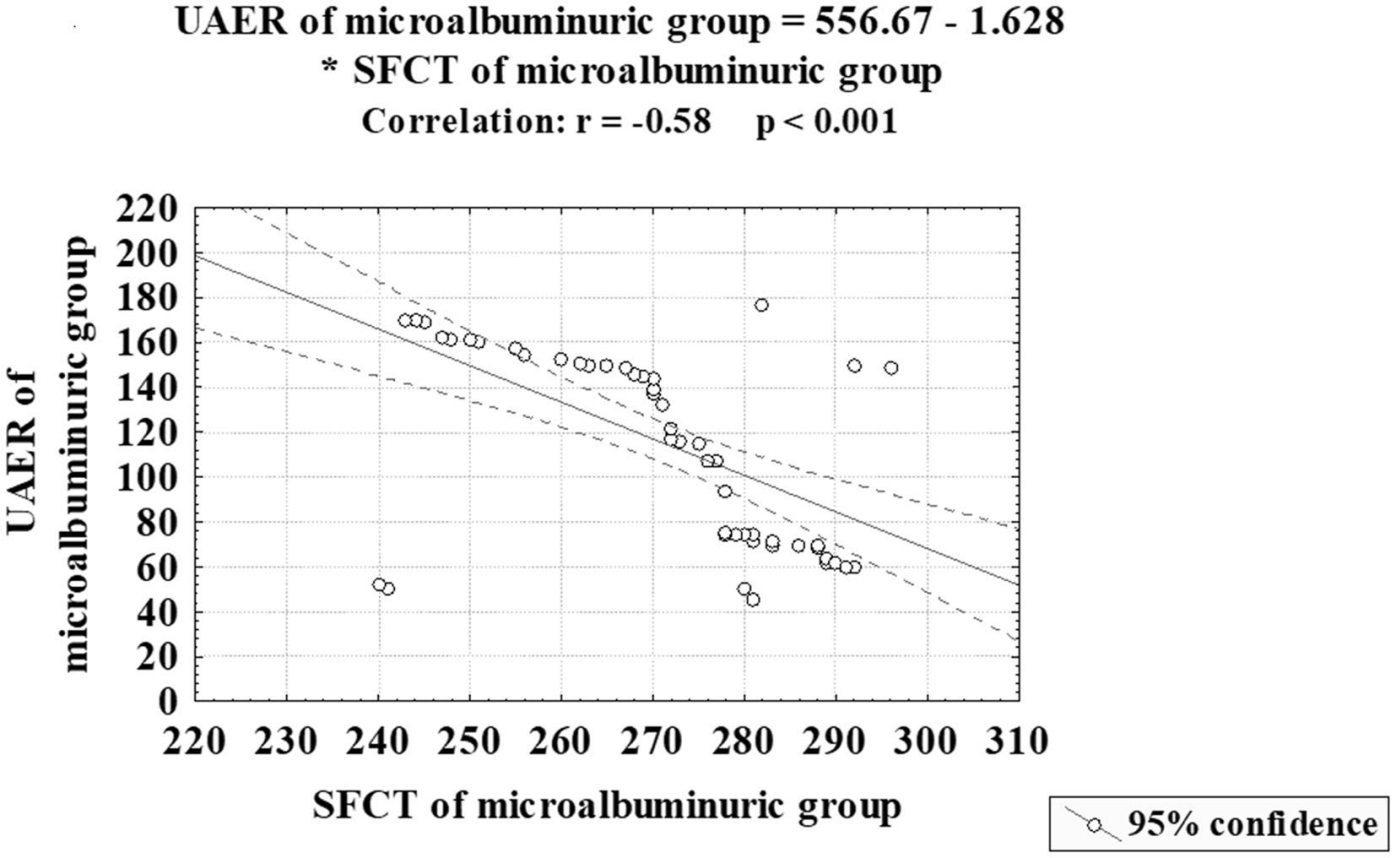
Correlation between subfoveal choroidal thickness in the innermost 1 mm circle and urinary albumin excretion rate in diabetic patients with microalbuminuria (Group 3). *SFCT* subfoveal choroidal thickness; *UAER* urinary albumin excretion rate

We compared SFCT in group 3 to the patients’ age, duration of DM, HbA1c, RBS, UAER, serum creatinine, eGFR, IOP, SE, and AL. In diabetic patients with microalbuminuria (Group 3), SFCT showed only a significant negative correlation with UAER (r  =  − 0.58 and p  <  0.001). (Table 3; Fig. 1).

**Table 3:** Correlations between subfoveal choroidal thickness (SFCT) and different parameters in the microalbuminuric group

	SFCT in microalbuminuric patients (Group 3)	
	r	p
Age (years)	− 0.08	0.543
Duration of DM (years)	− 0.20	0.125
HBA1c%	− 0.23	0.077
RBS (mg/dl)	− 0.07	0.595
IOP (mm Hg)	− 0.24	0.065
SE (D)	− 0.05	0.704
AL (mm)	− 0.09	0.494
UAER (µg/min)	− 0.58	< 0.001
Serum creatinine (mg/dL)	0.08	0.543
eGFR (mL/min/1.73 m^2^)	− 0.01	0.940

*HBA1c* glycosylated hemoglobin; *RBS* random blood sugar; *CCT* central corneal thickness; *IOP* intraocular pressure; *SE* spherical equivalent; *AL* axial length; *UAER* urinary albumin excretion rate; *eGFR* estimated glomerular filtration rate

Also, CT in the 3- and 6-mm rings (8 sectors) in microalbuminuric patients (Group 3) showed a significant negative correlation with UAER (Table 4).

**Table 4:** Correlations between HBA1c% and urinary albumin excretion ratio (UAER) with the choroidal thickness (CT) in different sectors in the microalbuminuric diabetic patients

Variable (µm)	Microalbuminuric group			
	HBA1c%		UAER (µg/min)	
	r	p	r	p
SFCT	− 0.23	0.077	− 0.58	< 0.001
SIM	− 0.18	0.169	− 0.69	< 0.001
IIM	− 0.04	0.762	− 0.55	< 0.001
TIM	− 0.08	0.649	− 0.60	< 0.001
NIM	− 0.15	0.146	− 0.65	< 0.001
SOM	0.02	0.704	− 0.39	0.002
IOM	− 0.07	0.879	− 0.35	0.006
TOM	− 0.15	0.253	− 0.56	< 0.001
NOM	− 0.06	0.649	− 0.53	< 0.001

*SFCT* subfoveal choroidal thickness; *SIM* superior of the inner macula (CT); *IIM* inferior of the inner macula (CT); *TIM* temporal of the inner macula (CT); *NIM* nasal of the inner macula (CT); *SOM* superior of the outer macula (CT); *IOM* inferior of outer macula (CT); *TOM* temporal of the outer macula (CT); *NOM* nasal of the outer macula (CT)

In multiple regression analyses, UAER was found to be the most important determinant of SFCT in patients with microalbuminuria (Table 5).

**Table 5:** Multiple regression analysis of different factors affecting subfoveal choroidal thickness (SFCT) in microalbuminuric patients

	Microalbuminuric patients			
	β	B	t	p value
Intercept		156.65	1.42	0.161
Age (years)	0.01	0.03	0.09	0.926
Duration of DM (years)	0.12	0.75	1.03	0.308
HBA1c%	− 0.16	− 1.93	− 1.32	0.193
RBS (mg/dl)	0.11	0.03	0.93	0.357
IOP (mm Hg)	0.17	1.99	1.48	0.146
SE (D)	− 0.06	− 2.41	− 0.54	0.589
AL (mm)	0.02	0.67	0.20	0.845
UAER (µg/min)	− 0.48	− 0.17	− 3.96	< 0.001
Creatinine (mg/dL)	0.16	21.86	1.54	0.1309
eGFR (mL/min/1.73 m^2^)	− 0.04	− 0.12	− 0.40	0.693

Regression summary for SFCT of diabetic patients with microalbuminuria R  =  0.641; R^2^  =  0.411; Adjusted R^2^  =  0. 0.306 F (9.50)  =  3.884p

*HBA1c* glycosylated hemoglobin; *RBS* random blood sugar; *CCT* central corneal thickness; *IOP* intraocular pressure; *SE* spherical equivalent; *AL* axial length; *UAER* urinary albumin excretion rate; *eGFR* estimated glomerular filtration rate

## Discussion

In the current study, we used SD-OCT to assess CT in diabetic patients with and without albuminuria in the absence of retinopathy. Although SS-OCT might be a preferred imaging modality for choroidal visualization owing to better penetration of deeper tissues by the longer wavelength sweeping laser [24]. However, with the more availability of SD-OCT as well as its ability to clearly visualize the sclerochoroidal interface using EDI (93% of 3468 subjects) as found by Wei et al. [25], SD-OCT was used in the current study. Failure of visualization is usually related to inadequate light penetration as in cases of media opacity [24]. Thus, all cases with media opacity or poor-quality OCT were excluded from this study. The same technique for choroidal thickness measurement was applied to a control group for standardized comparison.

The mean value of CT in diabetic patients with microalbuminuria was significantly lower compared to controls in the 9 ETDRS sectors. A decremental pattern of the CT of the nine locations was noticed, being the lowest for microalbuminuric ones (Group 3).

The relationship between DR and UAER was previously studied. Microalbuminuria was found as a predictor of DR. Moreover, greater prevalence of DR and more severity were reported in patients with macroalbuminuria than in those with microalbuminuria [21, 26]. While there is limited research on the relation between CT in diabetic patients and UAER. To the best of our knowledge, this relation was studied twice before. Like our results, Farias et al. found that choroidal changes were present in type 2 diabetic patients (37 patients) before the clinical development of retinopathy. Microalbuminuria was significantly correlated with a reduction in choroidal thickness and volume in diabetic patients without retinopathy [28]. On the contrary, Oliveira-Ferreira et al. [18] in their retrospective study stated that the SFCT was higher in diabetic patients without DR when compared to nondiabetic patients. Microalbuminuria was accompanied by thickening of the subfoveal and temporal choroid in diabetic patients without DR when compared with normoalbuminuric diabetics and non-diabetics [18]. These controversial results might be attributed to the manual measurement of CT in specified points rather than obtaining a map. Additionally, the effect of circadian rhythm may be a contributing factor. Both studies have not included the renal function tests of the patients. The included sample in the current study had normal serum creatine and eGFR thus none had chronic diabetic kidney disease [27]. The current study was designed to allow matched characteristics between the study groups and avoid the limitations of previously published research.

Another study on a relatively small sample of type 1 diabetic patients was done by Malerbi et al. [29]. They found that microalbuminuria was associated with increased choroidal thickness in type 1 diabetes mellitus without diabetic retinopathy. This difference between type 1 and type 2 DM is not well understood however, it could be related to the different age groups included in the studies, difference in disease duration, or could be related to the insulin resistance encountered in type 2 but not in type 1. Further longitudinal comparative studies are needed to clarify these differences.

In our study, CT in the 9 ETDRS sectors in diabetic patients with microalbuminuria showed no significant correlation with age, duration of DM, HbA1c, RBS, IOP, SE, AL, serum creatine, or eGFR. Similarly, Abadia et al. did not show any statistically significant relationship between HbA1c or the duration of diabetes and CT [30].

Choroidal thickness showed a negative correlation with UAER. This agrees with Farias et al. and Kocasarac et al. who found a significant negative correlation between proteinuria and CT in diabetic patients with nephropathy [19, 28]. On the contrary, Garrido-Hermosilla et al. [31] found a significant positive correlation between CT and albuminuria in their study in which patients with clinically evident DR were included.

Aiming to understand the effect of different variables on CT, we performed a multi-regression analysis, which showed that UAER is an important determinant of SFCT in microalbuminuric patients.

Variability in the results of the published studies highlights the possibility of the involvement of variable pathophysiological mechanisms. The increase in CT noticed in different studies was suggested to be a result of increased release of vascular endothelial growth factors (VEGF) or other cytokines, which mediate choroidal vasodilation and increased choroidal blood flow. Consequently, these changes may lead to an increase in the choroidal vascular layer thickness or increased vascular permeability with the consequent choroidal swelling [15, 32].

It has also been noted in several articles that the increased dropout of choroidal vessels may explain choroidal thinning, loss of choroidal capillaries, increased vascular resistance, and decreased choroidal flow in the foveal region. With the aid of Doppler flowmetry, choroidal blood flow and volume were found to be reduced in the foveal region in diabetic patients, including those without retinopathy [33, 34, 28, 29].

Kocasarac et al. [19] suggested that DM could affect CT through various mechanisms, such as autonomic dysfunction and microvascular damage. It may increase or decrease CT according to the most influential pathological effect. This vascular involvement has been supported by Sousa et al. [35] who demonstrated an early impairment of the physiological retinal vascular response in patients with type 1 DM without clinical diabetic retinopathy.

In our study, although statistical analysis yielded significant results, however, we still believe that the results cannot be generalized. Owing to the controversial results in the literature, including our study, multicenter studies utilizing different designs and follow-up periods may provide more conclusive and consistent results. It remains unclear whether diabetic choroidopathy is a predicting, modulating, or causative factor of DR.

The current study is limited by the relatively small sample size, lack of follow-up, and the manual outline of the sclerochoroidal junction. In addition to being done in a single center with inclusion of patients from same ethnicity.

## Conclusions

Decreased CT was significantly correlated with UAER in diabetic patients without retinopathy and otherwise normal kidney functions. This decrease in thickness might be a predictor of DR. Further longitudinal studies are needed for a definite conclusion.

## Data Availability

Data supporting the results reported in the article can be accessed after communication with the corresponding author.
